# Use of nanoparticles in animal models for prostate cancer treatment: a systematic review

**DOI:** 10.1590/acb385923

**Published:** 2023-10-30

**Authors:** Michele Selzler, Alexandre Moreira de Almeida, Marcelo Barbosa Neves, Alessandra de Figueiredo Gonçalves, Ricardo Dutra Aydos, Rondon Tosta Ramalho

**Affiliations:** 1 Universidade Federal do Mato Grosso do Sul – Postgraduate Program in Health and Development in the Midwest Region – Campo Grande (MS), Brazil.; 2 Universidade Federal do Rio de Janeiro – Postgraduate Program in Biological Sciences – Rio de Janeiro (RJ), Brazil.

**Keywords:** Nanoparticles, Nanomedicine, Prostatic Neoplasms

## Abstract

**Purpose:**

To conduct a systematic review of nanoparticles’ use in the treatment of prostate cancer in animals.

**Methods:**

A systematic review was conducted in the databases PubMed, Scientific Electronic Library Online (SciELO), Latin American and Caribbean Health Sciences Literature (LILACS), Cochrane Library, and EMBASE, and the descriptors were chosen based on terms indexed in Health Sciences Descriptors (DeCS)/Medical Subject Headings (MESH), which are: nanoparticles, nanomedicine, and prostate cancer. The systematic review protocol was registered in the International Prospective Register of Systematic Reviews (PROSPERO) with ID CRD42021271008.

**Results:**

A total of 3,897 articles was chosen; after reading the inclusion and exclusion criteria, six scientific articles with themes involving nanoparticles carrying medications were reached. Among the nanoparticles found, there were carboxymethylcellulose polymer, micellar casein nanoparticles, liquid crystal nanoparticles, serum albumin nanoparticles, and poly(ethylene glycol)-block-polylactide (mPEG-PLA) conjugated nanoparticles encapsulating cabazitaxel, docetaxel, and flutamide, which were nanoparticles used to treat prostate cancer in animals.

**Conclusions:**

Through using nanoparticles to encapsulate medications for treating prostate cancer in animals, studies show a decrease in weight and tumor reduction, with nanoparticles resulting in greater survival time than free medications. The improved permeability and retention effect of nanoparticles in the bloodstream contribute to their effectiveness.

## Introduction

Cancer is a major public health issue; in 2018, approximately 18 million new cases of cancer were registered worldwide^1^. Prostate cancer (PCa) is the fifth leading cause of cancer death worldwide, with rates of 375,304 deaths per year, and the incidence rate in 2020 was 1,414,259 new cases of PCa worldwide^2^.

PCa is a solid tumor that has permeable tumor vessels due to irregular neovascularization, which facilitates the treatment and targeting of drugs with nanoparticles (NPs) due to the permeability and retention effect (PRE). NPs with sizes of 100–200 nm are ideal for reaching solid tumors by PRE without being eliminated, and they also exhibit fewer adverse effects and better therapeutic responses when compared to conventional chemotherapeutics^3^.

Taking into account technological advancement and tumor treatment alternatives, nanoparticle-based targeting and delivery systems have demonstrated beneficial effects in cancer treatment^4^.

The use of NPs in the development of effective actives for biological targets with controlled release increases bioavailability without changing distribution or absorption. Efficiently developed formulations can reduce toxicity risks, and treatments can be administered via a variety of routes, including oral consumption, inhalation, intravenous, and subcutaneous injection^5^.

The proposal was to conduct a systematic review based on in-vivo animal studies on the use of medications and NPs in the treatment of PCa in order to improve existing studies and provide new insights for clinical studies.

## Methods

The systematic review protocol was registered in the International Prospective Register of Systematic Reviews (PROSPERO) with ID CRD42021271008, and the research design was carried out according to Review of Data from Experimental Studies in Animals (CAMARADES)^6^ and the Preferred Reporting Items for Systematic Reviews and Meta-Analyses (PRISMA) protocol recommendations.

This review was created and carried out to answer the following question: what are the advantages of using NPs and drugs in the treatment of animal prostate cancer? The PICO strategy was used to formulate the question, as follows:

- P: animals (rats and mice) inoculated and induced for prostate cancer;
- I: treatment with NPs and medication;
- C: animals given free medicine and saline solution;
- O: Primary outcome: efficacy of treatment using NPs and secondary outcomes–tumor regression, animal weight loss, underlying organ toxicity.

Based on the question and the PICO strategy, the inclusion and exclusion criteria were developed. The criteria for inclusion was: scientific papers published in any language that described the use of drugs and NPs to treat PCa in animals (male rats and mice). The criteria for exclusion were:

- Pre-2010 publications;
- Literature reviews, letters, case reports, theses, book chapters, abstracts, in-vitro studies, and human studies;
- Herbal medicine studies;
- PCa diagnosis studies.

The databases used for the research were Medical Literature Analysis and Retrieval System (MEDLINE), Scientific Electronic Library Online (ScieLO), Latin American and Caribbean Literature in Health Sciences (LILACS), and Virtual Health Library (BVS Brasil). Nanoparticles, nanomedicine, and prostate cancer were used as descriptors.

The initial search strategy included the following different terms: “Nanoparticle”, “Nanomedicine”, “Prostate cancer”, “Cancer”, “Prostatic Neoplasms”, “Nanoparticles AND Prostatic Neoplasms”, “Nanoparticle OR Nanomedicine AND Prostatic neoplasms”, “Nanomedicine OR Nanoparticle AND Prostate Cancer”, “Nanotherapy OR Nanoparticle AND Prostate Cancer”, “Nanoparticle OR Nanotherapy AND Prostate Cancer”, “Nanoparticle OR Nanomedicine AND Prostate Cancer”, “Nanoparticle OR Nanotherapy AND Prostate AND CANCER”, “Nanoparticle OR Nanotherapy AND Prostate CANCER”, “Nanoparticle AND Prostate AND Cancer” e “Nanoparticle AND Prostate Cancer”.

The Intelligent Systematic Review (RAYYAN) tool was used to assist researchers in screening and selecting articles for study selection. Based on the inclusion and exclusion criteria, two researchers independently analyzed the selected articles. Duplicate articles were excluded in the first phase, and titles and abstracts were read individually and blindly. The articles were read in full in the second phase, and the differences in study selection were discussed between both.

To assess the risk of bias in each study, the Systematic Review Center for Laboratory Animal Experimentation (SYRCLE) tool was used. This tool was adapted from the Cochrane Collaboration RoB Tool, which evaluates randomized controlled trials. The SYRCLE tool was modified to accommodate randomized controlled trials and interventions in animal studies. The tool includes 10 entries on selection bias, performance bias, detection bias, and attrition bias. Answers to judgments are “yes” for low risk of bias, “no” for high risk of bias, and “unclear” for insufficient data. Two researchers answered the questions independently, and there were no disagreements.

The methodology used in the studies was heterogeneous, and it was not possible to determine the relative data of each study; thus, no data meta-analysis was performed.

## Results

**Figure 1:**
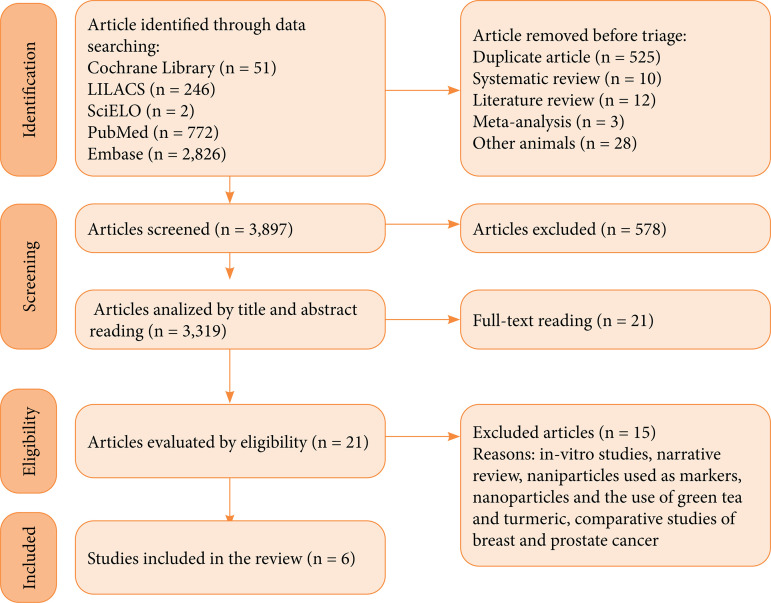
Identification of studies through database searching.

Source: Elaborated by the authors.

There were 3,897 articles chosen, including 51 from the Cochrane Library, 246 from Lilacs, two from SciELO, 772 from PubMed, and 2,826 from EMBASE. Before screening, duplicate articles (n = 525), systematic review articles (n = 10), literature review articles (n = 12), meta-analyses (n = 3), and other articles that researched animals other than the focus of the systematic review, such as dogs (n = 11), swine (n = 7), fish (n = 6), and rabbits (n = 4), were excluded. There were 3,319 articles in the screening selection. Following the inclusion and exclusion criteria, 21 articles were read in full after reading the titles and abstracts. The NPs and medications used in these studies were actively directed, linked to receptors, or even natural treatments unrelated to the proposed theme, so 15 articles were excluded, leaving six articles for the study (Fig. 1).

**Figure 2:**
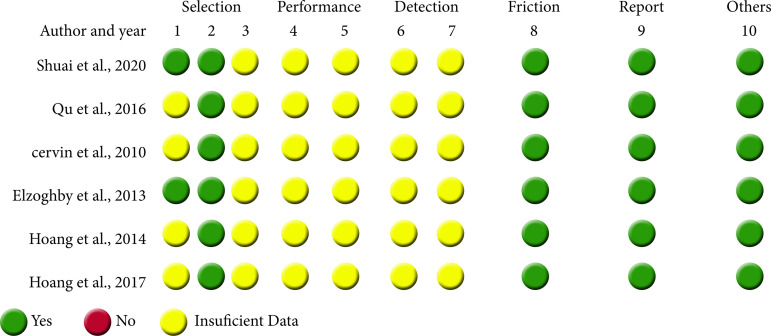
Risk of bias in individual studies (SYRCLE Rob toll criteria). YES responses indicated low risk of bias, NO indicated high risk of bias, and insufficient data indicated unable to attribute bias.

Source: Elaborated by the authors.

Figure 2 presents the results of the risk of bias assessment for each included study. Only two authors reported the sequence of animal allocation and the characteristics of the animals in treatment and control groups, according to the studies; in the other studies, there was no information about the allocation of the different groups, no information about the housing of the animals, no information about whether the experiment was performed at random, no information about whether the investigators were blinded to the intervention received, and no information about the treatment received. All studies included data on the findings of the experiments, and none of them presented issues that could have resulted in a high risk of bias. There were no high-risk or methodological restrictions that forced the exclusion of publications chosen for this systematic review.

The selected studies’ characteristics were consistent with the chemotherapeutics utilized; three trials used cabazitaxel (Cbz), two used docetaxel (DTX), and one used flutamide (FLT). All medications were nanoparticle-coated with the goal of treating rats and mice generated with PCa or those with drug resistance. All the developed NPs had diameters larger than 5 nm and less than 200 nm. The size limit of the NPs allows them to target tumors through permeability and retention effects, and they were given intravenously in most of the included studies, except for one, which compared the use of taxotere and liquid crystal nanoparticle (LCNP)/DTX intraperitoneally and intravenously. Table 1 describes the animals utilized in the study, cell lines, the interventions, and the outcome.

In terms of experimental design, the animal model studies were heterogeneous due to the medication employed, the administered dose, the duration of the investigation, and the induction of PCa in the animals. Four of the six selected studies observed tumor growth after injecting PC-3 PCa cells subcutaneously into the flanks of animals. To assess drug resistance, one study inoculated Du-145 human PCa cells subcutaneously into the flanks of healthy ICR mice, and two studies inoculated PC-3 cells and C4-2BRES cells directly into the bone marrow. In another study, the hormone androgen was used to induce PCa in animals, and prostate-specific antigen (PSA) levels were measured to substantiate the presence of the protein in the animals’ blood.

The studies showed that the drugs carried by the NPs released more slowly and had a greater capacity for accumulation in tumors. During the treatment period, the average tumor size and weight of the animals were monitored, and reduction in tumor size was observed with the use of NPS and medications. Among the six articles selected, four examined the antitumor activity and the risks of adjacent organ toxicity, with the liver being the most reported. It was also observed that the animals in the control group lost less weight than those receiving free medication.

DTX was used in combination with two different NPs (carboxymethylcellulose–Cellax and LCNP) in two models of PCa (mice inoculated with PC-3 resistant and another model inoculated with PC-3), only one dose of Cellax/DTX completely regressed a castration-resistant PCa in 120 days, demonstrating low adverse effects and drug resistance in addition to increasing survival in cases of metastasis. Both native DTX-treated mice and control mice exhibited bone stasis. Similarly, when analyzing the mean tumor volumes, LCNP/DTX demonstrated a superior effect on tumor regression compared to the free drug.

Taxotere was administered intraperitoneally and intravenously in conjunction with LCNP. When administered intraperitoneally, LCNP/taxotera demonstrated less antitumor activity than when administered intravenously, which effectively decreased tumor volume. In contrast, FLT associated with casein (CAS) micelles demonstrated superior antitumor activity in an androgen-dependent model of PCa induced with cyproterone acetate and testosterone, reducing tumor proliferation, controlling tumor angiogenesis, and increasing tumor apoptosis with decreased hepatotoxicity compared to free medication.

**Table 1:** Presentation of selected studies.

Study	Population			Intervention					OUTCOME
	Cell lines	Animal model	Nanoparticle		Medication (administered route)	Dose (mg/kg)	Size of NPs		
Shuai et al.^10^	DU145	BALB/c nude mice (4-5 weeks old), five groups (n = 7). 5 groups (n = 10) of ICR mice (4-5 weeks old)	Self-assembling poly(ethylene glycol)-block-polylactide-cabazitaxel		Cabazitaxel (IV)	8 15 30	20–30	During the treatment investigation, administration of the mPEG_4k_-PLA_4k_- Cbz conjugate nanoassembly at a reasonable dose resulted in long-lasting tumor regression. Notably, mPEG_4k_-PLA_4k_-Cbz nanotherapy has been shown to significantly reduce animal toxicity compared to that of the drug’s free form. Therefore, in terms of in-vivo anticancer efficacy and safety profiles, mPEG_4k_-PLA_4k_-Cbz nanotherapy fared better than other polymeric nanotherapies.	
Qu et al.^13^	PC-3	BALB/c nude mice (6 weeks old): three groups(n = 6).	Human serumalbumin		Cabazitaxel (IV)	8	110–140	Comparing Cbz-NPs versus Cbz-Tween reveals considerable safety gains. In the in-vitro hemolysis assay, superior blood biocompatibility was shown. Along with decreased toxicity, Cbz-NPs demonstrated increased Cbz accumulation in tumors and longer blood circulation. As a result, Cbz-NPs may one day be used in the treatment of prostate cancer	
Cervin et al.^15^	PC-3	Male SCID mice (4–6 weeks old) Study 1: 30 mice were divided into three groups (n = 10). Study 2: 18 mice were divided into two groups (one group of 12 and one group of six animals).	Liquid crystal nanoparticle (LCNP)		Study 1: Docetaxel (IV) Taxotere (IP) Study 2: Docetaxel (IV) Taxotere (IV)	1.62 1.35	80–90	Compared to the docetaxel formulation, the LCNP/docetaxel formulation had a better effect on tumor regression. In studies 1 and 2, it was demonstrated that LCNP/docetaxel had an effective antitumoral impact. The effect was substantial and nearly equal between the two experiments, despite some variations in the administration schedule and a 20% lower dose in study 2. In study 2, statistical variations in tumor volumes were identified. Along with the 20% lower dose, the different routes of administration can help to explain why taxotere had a less potent antitumoral impact in study 1 than it did in study 2	
Elzoghby et al.^17^	Androgen-dependent PCa induced with cyproterone acetate and testosterone	Sprague-Dawley rats divided into three groups of eight rats	CAS micelles loaded with FLT		Flutamide (IV)	12	100	In-vivo evaluation of FLT-CAS micelles found that micellar FLT had better anti-tumor efficacy than free drug, as seen by a decrease in PSA serum level, prostate and seminal vesicle relative weights, and histological alterations. Furthermore, FLT-CAS micelles were reported to effectively reduce prostate tumor cell proliferation, limit tumor angiogenesis, and improve tumor apoptotic induction. Hepatotoxicity was also reduced in drug micelles compared in free medications	
Hoang et al.^19^	PC-3	BALB/c mice were divided into three groups (n = 5) by animals. NOD/SCID mice were divided into three groups (n = 10) by animals	Carboxymethyl-cellulose		Docetaxel (IV)	170 20 10	122	One dose of Cellax totally regressed *s.c*. tumor xenografts in a mouse model of CRPC, demonstrating the drug’s improved effectiveness against the disease. Furthermore, it was discovered that Cellax caused fewer adverse effects than native DTX. In contrast to the original medication, Cellax did not cause the expression of drug resistance molecules to rise in androgen independent PC3 prostate cancer cells. When treated with Cellax vs native DTX and control, there were two- to three-fold increases in survival and notable improvements in the quality of life for the animals in the model of prostate cancer metastases to the bone	
Hoang et al.^20^	PC-3	NOD/SCID mice were divided into three groups (of five) based on animal	Carboxymethyl-cellulose		Cabazitaxel (IV)	55	100	Except for RES tissues, Cellax- Cbz showed lower accumulation in most normal organs while having a 157-fold increase in tumor delivery relative to free Cbz. When compared to free Cbz, Cellax-Cbz demonstrated greater anti-tumor action in a dose-dependent manner against models of DTX-resistant CRPC. It is noteworthy that 70% of mice with bone metastasis and DTX-resistant PC received a long-lasting cure (> 120 days) from Cellax-Cbz, whereas animals treated with saline or free Cbz died from the same disease after a month	

Cbz: cabazitaxel; CAS: casein; FLT: flutamide; DTX: docetaxel; CRPC: castration resistant prostate cancer; RES: resistant. Source: Elaborated by the authors.

Cbz was combined with three different NPs (mPEG_4k_-PLA_4k_-Cbz, Cbz-NPs, and Cellax-Cbz) and tested in three different xenographic models (mice inoculated with DU145, PC-3, and PC-3 resistant), demonstrating an increase in tumor development inhibition and toxicity reduction. When combined with Cellax (Cellax-Cbz), it demonstrated a remarkable ability to direct drug release directly into the tumor with minimal accumulation in normal tissues, thereby increasing the survival of mice with PCa and bone metastases, even in chemotherapy-resistant tumors.

## Discussion

Polylactic acid (PLA) and poly(ethylene glycol) (PEG) are the most commonly used synthetic polymers. Both are used together (MPEG-PLA), and, when combined with other polymers, they produce remarkable molecular carriers that can be administered in aqueous or lipid media^7^. Amphiphilic block copolymers (a hydrophilic polar part and a hydrophobic non-polar part) are used to transport hydrophobic drugs like CTX, allowing them to stay in the bloodstream longer, reducing drug release, and ultimately reaching the tumor for drug delivery^8^.

The studies that used NPs conjugated to PEG obtained promising results in directing the NPs into target cells without exposing other tissues or going through the endothelial reticulum system’s opsonization mechanism^9^. In one selected article, the experiments using preclinical models of PCa showed that the use of prodrug nanotherapy associated with PEG and PLA amphiphilic polymers to maintain pharmacological activity and reduce adverse effects was beneficial. Chemotherapy’s slow-release limited drug exposure to tissues and other organs, in contrast to free drugs, which, in addition to reaching the tumor, also affected the tissues and thus had side effects^10^.

The same NPs of MPEG-PLA encapsulating cisplatin were evaluated in a study for the treatment of ovarian cancer, observing the toxicity presented by the animals due to the use of different doses. The mortality rate of the animals treated with NPs and cisplatin at 100 mg/kg was 40%, and, when compared to the use of free cisplatin at 10 mg/kg, it was 62.5%. As a result, using NPs loaded with cisplatin at higher doses is safer for clinical use^11^.

Albumin has the highest concentration of plasma proteins in the body and, due to its high solubility, it is an excellent drug carrier for tumor cells. DTX-NPs constructed by using human serum albumin (HSA) demonstrated that these NPs can offer promising anti-tumor activity with low systemic toxicity^12^. This fact was also verified in our results through the study of Qu et al.^13^, who used HSA to construct Cbz-NPs, revealing considerable safety gains and superior blood biocompatibility; along with decreased toxicity, Cbz-NPs demonstrated increased Cbz accumulation in tumors and longer blood circulation.

In most preclinical studies, the intravenous route is the preferred method of chemotherapy administration. Bioavailability is measured by the percentage of the drug that is absorbed in the body, and this information can be used to determine the appropriate doses for treatments and characterize the benefits and side effects that the drug has on the body^14^.

Our results showed one preclinical study in which effective encapsulation LCNP with stable drug release were developed. Two separate studies were conducted, with study 2 correlating the findings of study 1. Both studies investigated the use of LCNP/DTX, as well as free taxotere. Taxotere, on the other hand, was administered intraperitoneally in study 1, as opposed to intravenously in study 2. Even when administered differently, free taxotere had a lower antitumor effect^15^. Because of its solubility and stability, the chemotherapy drug DTX has some limitations in the treatment of tumors. Thus, it is estimated that research is being conducted on the development of new drugs that are less toxic to the body and more effective at penetrating and directing chemotherapy to tumor tissues^16^.

A nanoparticle prepared from CAS, a milk protein that facilitates drug release due to its amphiphilic property, can involve different drug properties, such as hydrophobic or hydrophilic drugs, in addition to being cheap and easily accessible, biodegradable, and non-toxic to the body^16^. One selected study showed that the use of CAS micelles loaded with FLT is justified because it is more effective and safer in inhibiting and binding dihydrotestosterone to the target cell receptor, which affects the plasma PSA, because this drug has low aqueous solubility, permeability, and hepatotoxicity^17^. In a previous study, hollow CAS NPs were prepared in an aqueous solution, and excellent tumor cell barrier penetration was observed^18^.

The use of drugs for long periods can cause high toxicity and resistance to treatment. With that in mind, two selected studies in this systematic review, with the intention of inhibiting tumor activity in mice resistant to castration, tested Cellax, a conjugate of polymer-DTX^19^. Subsequently, a new study tested these NPs, but through Cbz in animals with PCa and bone metastases resistant to DTX, now with the aim of directing the drug to the tumor while preserving the organs of the treated animals^20^. This is supported by the literature, which demonstrates that intravenous administration of NPs and drugs provides greater access to body tissue, increasing the potential and likelihood of reaching the biological destination. The NPs that encapsulated the drugs remained in circulation for a longer period of time before reaching the tumor tissue via the PRE in all of the studies. The size of the NPs interferes with tumor targeting. NPs with sizes less than 200 nm are easier to target tumors passively using the PRE, which is more effective than the free drug^21^.

In general, when compared to free drugs, all studies that passively directed drug-bound NPs to tumor tissue via the PRE demonstrated significant therapeutic efficacy. This is possible because chemotherapy drugs with low molecular weight are rapidly eliminated by renal glomerular filtration. The use of nanocarriers in combination with chemotherapeutics extends the pharmacokinetics and, as a result, increases bioavailability. When incorporated drugs are used, animal survival and tumor reduction are higher than when free drugs are used^22^.

We can observe the effectiveness of NPs in different treatments, as seen in three studies selected for this systematic review, in which the polydispersity index (PDI) was used to evaluate particle uniformity, required due to the results of cell uptake by endocytosis. The PDI results were favorable for all the researchers’ NPs developed for the treatment of PCa, with an index close to 0^10^^,^ 19 ^,^ 20. This is consistent with a study in mice with brain tumors using menthol-modified CAS NPs as a treatment that obtained PDI values of NPs close to 0 with effective values of 0.20±0.1 PDI^23^.

Numerous doses of chemotherapy are used to treat cancer, as long as there is no toxicity due to the maximum tolerated dose. Pre-clinical studies can confirm the link between drug doses and tumor reduction and weight loss in animals. Determining the maximum tolerated dose in preclinical studies aids in the evaluation of the initial doses of treatment used in humans while taking side effects into account^24^. The finding is also consistent with our review, which selected two studies using Cellax to treat PCa in animals and demonstrated that, even at different doses, animals treated with Cellax did not lose body weight, in addition to showing no changes in hematological parameters in animals treated with Cellax, and that the use of larger encapsulated doses can be used to treat PCa without harming other organs and tissues^19^^,^ 20.

Carboxymethylcellulose-based NPs loaded with DTX were employed in in-vivo models for the treatment of breast cancer, yielding promising outcomes. The administration of free DTX was conducted at the maximum acceptable dose, but Cellax-DTX was delivered at a dosage three times greater, resulting in notable efficacy in the treatment. Specifically, Cellax-DTX exhibited a 90% suppression of tumor development. The researchers reached the conclusion that, like the treatment of PCa, the administration of higher doses of chemotherapy medications can be employed in a safe manner for the treatment of mice afflicted with breast cancer^25^. The findings presented are in alignment with our comprehensive analysis of literature review, in which three articles demonstrated that tumor activity in the subjects under treatment was assessed through tumor regression and loss of body weight. The administration of saline solution led to a swift escalation in tumor growth, whereas the application of free medication resulted in substantial reduction of tumor size.

Notably, the most favorable outcomes in terms of tumor disappearance were observed when utilizing NPs in conjunction with chemotherapy^10^^,^ 13 ^,^ 15. In the realm of animal research focused on lung cancer treatment, the utilization of HSA-NPs with DTX has demonstrated notable therapeutic effects, surpassing the efficacy of free DTX. In addition, it was observed that NPs had an extended presence in the circulatory system, leading to enhanced permeability and retention^12^.

The efficacy of a combination of cisplatin and DTX encapsulated with LCNP linked to folate in targeting cancer was evaluated in a study for the treatment of breast cancer with bone metastases^26^. Their results were similar to those reported in articles using PCa studies: the animals treated with the free drug lost body weight, unlike the animals treated with nanoparticulate medication, which did not lose body weight during the study. Furthermore, these animals treated with medication and NPs had a 100% survival rate. These outcomes are possible because of the longer storage time of encapsulated medications, which results in a reduction in adverse effects^15^^,^ 19.

Different formulations of NPs encapsulating hydroxycamptothecin modified with menthol were used in the treatment of mice with glioma and compared to the use of free medication. The results were also promising when encapsulated medications were used, with animals surviving 1.5 times longer when treated with CAS and menthol NPs. The organs (heart, spleen, liver, lung, and kidney) were removed and stained with hematoxylin and eosin for histological analysis, and no significant damage was found. According to the researchers, the menthol-linked nanocarriers have excellent tumor penetration capacity and low systemic toxicity, extending the average survival of the mice in the study^23^.

When female mice with metastatic breast carcinoma were treated with Cellax (170 mg/kg DTX) versus abraxane (170 mg/kg paclitaxel), 85% of mice treated with abraxane developed pulmonary metastasis, while 40% of mice treated with Cellax had some pulmonary nodules. Furthermore, abraxane treatment resulted in severe toxicity with metastasis (bone, spleen, liver, intraperitoneal space, and kidney), whereas Cellax treatment resulted in no metastasis^27^. A similar pattern emerged in selected studies by our review: with the animals maintaining a healthy body weight, the acute toxicity was negligible, revealing a decrease in drug levels in the heart, spleen, and kidneys^8^^,^ 13 ^,^ 17 ^,^ 20, demonstrating how NPs can be safe and effective.

During conducting this systematic review, some limitations were discovered. There are few clinical trials involving the use of NPs and chemotherapy for PCa treatment; additionally, there was heterogeneity in the studies used; the NPs had different properties; the dose concentrations and administration times were not the same; and these variations hampered the performance of a meta-analysis.

By considering the 3Rs in the context of this systematic review, researchers can promote ethical practices and foster a more responsible and compassionate approach to animal studies in the field of PCa treatment using NPs. In addition to assessing the efficacy of nanoparticle treatments and identifying the elements that lead to good outcomes using fewer animals in research without sacrificing the validity of the results, researchers can improve their experimental techniques to increase animal welfare and minimize any potential harmful effects on animals.

## Conclusion

This systematic review highlights the advantages of using NPs to encapsulate medications for treating PCa in animals. Studies show a decrease in weight and tumor reduction, with NPs resulting in greater survival time than free medications. The improved permeability and retention effect of NPs in the bloodstream contribute to their effectiveness.

## Data Availability

The data will be available upon request.
